# Human Selection

**Published:** 1913-01

**Authors:** 


### Department of Eugenics.

Conducted by Dr. Malone Duggan and Dr. Theo. Y. Hull.

### THE TEXAS STATE SOCIETY OF SOCIAL HYGIENE.

Headquarters: San Antonio, Texas.

Dr. Malone Duggan, President, San Antonio.
Dr. F. E. Daniel, 1st Vice President, Austin.
Prof. A. Caswell Ellis, 2d Vice President, Austin.
Mrs. Eli Hertzberg, 3d Vice President, San Antonio.
Dr. Theo. Y. Hull, Secretary, San Antonio.
Mr. W. L. Evans, Treasurer, San Antonio.

EXECUTIVE COMMITTEE.

Dr. Malone Duggan, Chm.
Dr. Theo. Y. Hull, Sec'y.
Dr. W. F. McCaleb.
Hon. C. H. Jenkins.
Rev. A. W. S. Garden.
Hon. J. C. Willacy.
Mr. W. L. Evans.
Dr. Frank D. Boyd.
Dr. J. R. Nichols.
Dr. J. M. McCutchan.
Dr. W. A. King.
Dr. J. W. Torbett.
Dr. F. C. Walsh.
Dr. Joe Reuss.
Dr. Frank Paschal.

### Human Selection.

It was said of the late Dr. Holmes that on one occasion, when called to see an epileptic child, he told the parents that a consultation should have been held fifty years before his visit. Dr. Holmes simply called attention to the fact that the child's condition was the result of parental defects handed down the ancestral line for one or more generations. Had the parents been free from certain defects, the child would not have been epileptic.

There is no doubt that the physical, mental and moral characteristics of the parents are inherited by the offspring. The known descendants of a certain woman addicted to alcoholism were 709. The mental and moral defects of this woman were distributed in the succeeding generations as follows: 100 illegitimate children, 181 prostitutes, 142 beggars, 46 workhouse inmates, 76 criminals and 164 more or less habitual drunkards. The cost to the State for the support of this family of criminals and defectives amounted to $1,200,000. Among fifty-seven children of alcoholics, only ten were normal physically and mentally.

Marriage is at once the most important and the most sacred of human relationships. The welfare of society depends far more upon the sacredness of this relationship and the characteristics of the parties entering it than it does upon the form of organized government. Beyond doubt, the most important factor in progress is human selection; but what kind of selection? There are those who lay great stress on natural selection. Since fully one-third of the human race dies before it reaches the age of selection, this might be termed microbic selection. Even Herbert Spencer felt constrained to issue the warning that modern hygiene and therapeutic methods interfere with the elimination of weaklings.

Every one agrees that the reproduction of weaklings should be discouraged. Microbic selection is very indiscriminate. Without doubt, had this kind of selection been so highly beneficial to the race as some would have us believe, the race should long ere this have reached a most exalted state of immunity. The rapid increase in the ratio of defectives in the last few decades and the immense drain upon the resources of the country therefrom does not indicate that natural selection is always beneficial.

Sooner or later the very nature of our civilization will compel the consideration of race culture from a higher viewpoint. Cupid, with his bow and arrow, has for many centuries left a trail of defectives and degenerates which a wiser selection could have obviated. Either Cupid must be taught to shoot a little more discriminately, or common sense must insist on an intelligent selection.

There is no other relationship into which human beings enter with so little thought. There is no other relationship from which so much is expected. The needs of the race at this present moment demand that fitness, physical, mental and moral worth, be the consideration in human selection. This is what Dr. Holmes meant.

